# Exercise improves behavioral dysfunction and inhibits the spontaneous excitatory postsynaptic current of D2-medium spiny neurons

**DOI:** 10.3389/fnagi.2022.1001256

**Published:** 2022-12-01

**Authors:** Gang Zhao, Danyu Zhang, Decai Qiao, Xiaoli Liu

**Affiliations:** ^1^Physical Education College, Soochow University, Suzhou, China; ^2^Physical Education and Sports College, Beijing Normal University, Beijing, China

**Keywords:** Parkinson’s disease, striatum, D2-MSNs, exercise, behavioral dysfunction

## Abstract

The abnormal function of striatal medium spiny neurons (MSNs) leads to the excitation-inhibition imbalance of the basal ganglia, which is an important pathogenic factor of Parkinson’s disease (PD). Exercise improves the dysfunction of basal ganglia through neuroprotective and neuroreparative effects, which may be related to the functional changes of expresses D2 receptors MSNs (D2-MSNs). In this study, D2-Cre mice were selected as the research objects, the PD model was induced by unilateral injection of 6-hydroxydopamine (6-OHDA) in the striatum, and the 4-week treadmill training method was used for exercise intervention. Using optogenetics and behavioral tests, we determined that the average total movement distance of PD and PD + Ex groups was significantly lower than that of the Control group, while that of the PD + Ex and PD + Laser groups was significantly higher than that of the PD group, and the two intervention methods of exercise and optogenetic-stimulation of the D2-MSNs had basically similar effects on improving the autonomic behavior of PD mice. To further investigate the cellular mechanisms, whole-cell patch clamp recordings were carried out on D2-MSNs. We found that exercise decreased the frequency and amplitude of spontaneous excitatory postsynaptic current (sEPSC) and increased the paired-pulse radio of D2-MSNs while leaving basic electrophysiological properties of MSNs unaffected. Combined with behavioral improvement and enhanced D2R protein expression, our findings suggest the inhibited sEPSC of D2-MSNs may contribute to the behavioral improvement after exercise.

## Introduction

Parkinson’s disease (PD) is a degenerative disease of the central nervous system characterized by progressive motor dysfunction. Insufficient activation of the substantia nigra-striatum dopaminergic (DA) pathway and hypertoxicity of the overexpressed cortical-striatum glutamatergic (Glu) pathway are considered responsible for the abnormal basal ganglia output in PD (Galvan and Wichmann, 2008; Perez-Ortega et al., 2016). DA fibers from the substantia nigra pars compacta, Glu fibers from the cortex, and medium spiny neurons (MSNs) in the striatum form a “triple” structure, which provides a special anatomical basis for the regulation of cortical-striatum Glu neurotransmission by DA (Brown et al., 2014).

Studies on the pathogenesis of PD have revealed that striatal expresses D2 receptors MSNs (D2-MSNs) are closely related to the development of PD (Baik et al., 1995; Anzalone et al., 2012; Lemos et al., 2016). DA depletion impairs the function of MSNs, leading to excess intracellular cations that cannot be pumped out and abnormally opened voltage-sensitive Ca^2+^ channels (Picconi et al., 2004). Therefore, the cortical-striatum synaptic transmission is enhanced, resulting in the overactivation of indirect pathways and dyskinesia (DeLong and Wichmann, 2015). Abnormal function of D2-MSNs may also alter the lateral inhibition of synaptic connections in the local loop of striatal MSNs, affecting the release and dispersion of neurotransmitters and enhancing the lateral inhibition of D1-MSNs and direct pathway activity (Descarries et al., 2008; Lopez-Huerta et al., 2013; Cazorla et al., 2014).

The neuroprotective effects of exercise can improve the function of the central nervous system (Cobianchi et al., 2017) and promote synaptic neural plasticity (Pin-Barre and Laurin, 2015). Regular physical activity can significantly reduce the risk of PD (Petzinger et al., 2010). Several studies have found that exercise can improve motor dysfunction in both PD patients and animal models (Zigmond and Smeyne, 2014; Hou et al., 2017; Jang et al., 2018), the mechanism of which may be related to the neuroprotective effects of exercise, which reduces damage to the substantia nigra DA neurons (Koo et al., 2017). Previous laboratory studies have confirmed that 4-week treadmill exercises activate the endogenous cannabinoid system and remodel cortex-striatum indirect pathway long-term depression (LTD) by upregulating the expression of the cortical-striatum postsynaptic membranes mGluR1/5 (Shi et al., 2019, 2021). This inhibits the excitotoxic effects of Glu overexertion on striatal MSNs, induces motion-dependent remodeling of striatal D2-MSNs dendritic spines, and improves motor dysfunction in PD animal models (Chen et al., 2020). Thus, striatal D2-MSNs are speculated to be an important cellular molecular target for exercise to improve basal ganglia dysfunction in PD. In recent years, optogenetics has been applied to neuroscience. In particular, neuropathological mechanisms using light-stimulated excitatory (channelrhodopsin-2, ChR2) and inhibitory (halorhodopsin, NpHR) light-sensitive proteins have been studied to achieve precise control of specific neuron function (Wentz et al., 2013). Optogenetic studies have confirmed that blue light-stimulated striatal D2-MSNs in transfected ChR2 normal mice could induce tremor and PD, and that stimulated striatal D1-MSNs could alleviate symptoms, such as tremor (Cui et al., 2013). To this end, this study aimed to use optogenetics technology to target striatal D2-MSNs and reveal the potential mechanisms underlying exercise-associated improvements in PD.

## Materials and methods

### Animals

**FIGURE 1:**
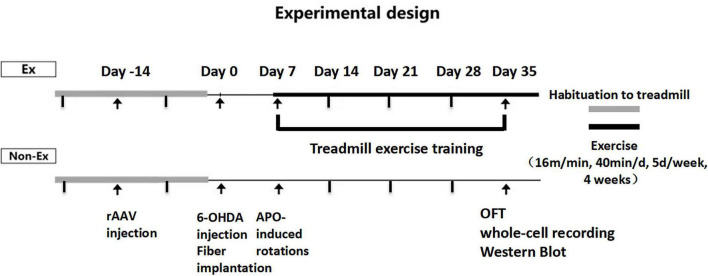
Experimental design.

Male D2-Cre mice (8 weeks old, Nation Key Laboratory of Cognitive Neuroscience and Learning, Beijing, China) were housed under artificial light (12 h light/dark cycle, lights on at 7:00 AM) at 22 ± 2 light/dark cycle, lights on at 7*ad libitum*. The D2-Cre male mice were randomly divided into three groups: Control group (Control, *n* = 6), PD group (PD, *n* = 6) and PD + Exercise group (PD + Ex, *n* = 6). The mice in each group were subjected to behavioral experiments in day 35 (Figure 1). The Animal Ethics Committee of Soochow University and Beijing Normal University Committee for Animal Care approved all experimental procedures, and this study complied with the guidelines set forth by the National Institutes of Health.

### Stereotactic injections of 6-hydroxydopamine and adeno-associated virus vectors

Each mouse was anesthetized with isoflurane (5%, 0.8 L/min) and then placed on a stereotaxic frame (Stoelting, Chicago, IL, USA). The viral vector carrying the NpHR (1 μl; rAAV-Ef1α-DIO-eNpHR-EYFP-WPRE-pA, titer ≥2.00 E + 12 vg/ml; Brainvta, China) was injected on the right dorsolateral striatum (bregma: AP, 0.5 mm; ML, 1.8 mm; DV, 3.0 mm) at a flow rate 0.1 μl/min, and the wound was sutured.

Two weeks after the adeno-associated virus (AAV) vectors were injected, PD and PD + Ex group mice were anesthetized with isoflurane (5%, 0.8 L/min) and fixed on a stereotaxic device. The skin was cut along the sagittal suture at the top of the skull, holes were drilled in the striatum (AP, 0.5 mm; ML, 1.8 mm), and 4 μl of 6-hydroxydopamine hydrobromide (6-OHDA) solution was injected (2 μg/μl with 0.02% ascorbic acid) at different depths of −3.0 and −2.0 mm. The needle was held for 3 min, and the sample was injected at a constant speed. Optic fiber (length: 2.5 mm; diameter: 200 mm; NA: 0.37; Thorlabs, Newton, NJ, USA) was placed right above the injection side. Optic fiber was anchored to the animal skull with a small screw and dental cement.

### Immunohistochemistry

Mice were anesthetized with isoflurane (5%, 0.8 L/min), perfused through the left ventricle-ascending aorta (30 ml saline and 30 ml 4% paraformaldehyde solution), and the brain tissue was quickly removed after perfusion. Fix in 4% paraformaldehyde solution for 24 h. The brain tissue was taken out, placed in a 30% sucrose solution, dehydrated, and then trimmed and embedded. The brain tissue was trimmed in a coronal plane and continuously coronally sliced to a thickness of 10 μm, 6 pieces each. Immunohistochemical staining, dehydration, transparency, mounting, primary antibody used: rabbit polyclonal antibody (1:1000, Abcam, USA), secondary antibody: anti-rabbit IgG (H + L) antibody (1:200, KPL, USA), fluorescence microscopy (DP72, Olympus, USA) photographed the dorsal region of the striatum. Statistical analysis of optical density (OD) of striatum tyrosine hydroxylase (TH) immunopositive fibers using Image-Pro Plus 6.0 software.

After the end of all patch clamp experiments, the mice striatum brain slices were fixed, dehydrated and embedded, and the virus transfection of the striatum brain region was observed under laser confocal microscopy.

### Western blotting

The expression level of striatal D2R was detected using western blotting. At the end of the 4-week exercise intervention, the mice were fasted for 24 h for sample collection after the behavioral test was completed. The damaged lateral striatum was peeled off and placed in liquid nitrogen for quick freezing. The tissue in liquid nitrogen was added to an appropriate amount of lysis buffer to extract the total protein of brain tissue in each group. The protein concentration was determined using the BCA method, which involved adding SDS buffer five times that was boiled for 5 min, cooled down, and then stored at −80°C refrigerator for testing. A 30 μg protein sample was taken, the protein was electrophoresed on a 12% SDS-polyacrylamide gel, and the protein was transferred to a polyvinyl difluoride membrane using the wet transfer method. Place in plastic wrap and incubate with blocking solution for 90 min. The membrane was blocked in 5% non-fat dry milk dissolved in TBST before being incubated overnight at 4°C with D2R primary antibody (1:1000, Abcam, Ab130295). The membrane was washed with PBS, and secondary antibodies were added, followed by shaking on a rotary shaker and incubated at room temperature for 90 min. The images were fixed and developed using chemiluminescence and analyzed using Image-Pro Plus 6.0 software, and the protein content was represented using the ratio of the gray value of the target protein to the gray value of the internal reference. When comparing different groups, the ratio of the target protein to the internal reference in the Control group was normalized to 100%, and the ratio of the remaining groups to the Control group average ratio represented the protein expression level.

### Exercise regimen and behavioral test

The exercise intervention program was uniform speed running (16 m/min, 40 min/day, 5 day per week for 4 weeks) (Hoydal et al., 2007). Control mice were exposed to the same environmental conditions (handling, treadmill motor noise) as exercised mice.

For PD model Identification, 7 days after 6-OHDA injection, PD model mice were injected with APO (0.5 μg/g) to induce rotational behavior, and the number of rotations of the mice within 30 min was recorded. The number of rotations >120 r/30 min is considered as the standard of the PD mice mode.

The autonomous activity of the mice was tested by Open Field Test (OFT). The open field experiment box is a 40 cm × 40 cm × 40 cm cube, and the four walls and the bottom are white. It is placed in a room with a light intensity of 20 Lux and no background noise. The digital camera is fixed at 80 cm directly above the box. Voluntary activities in the open field were recorded for 10 min. The indicators analyzed by Smart 3.0 software include: distance of motion distance, speed of movement and activity, namely: fast moving state (speed >15 cm/s); rest state (speed <5 cm/s); slow moving state (5 cm/s ≤ speed ≤ 15 cm/s).

**FIGURE 2:**
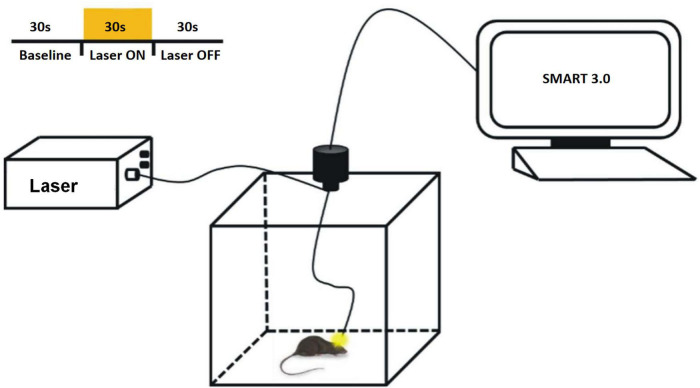
Optogenetic experiments design.

In the open field experiment of light-inhibited D2-MSNs in mice, the light source controller of optogenetic equipment (Shanghai Laser and Optics Century Co., Ltd.) was used to stimulate the mice at a wavelength of 589 nm and 10 mW of light (Figure 2). The stimulation program was a cycle of 30 s light, 30 s off, and 30 s interval, repeated 7 groups for a total of 10 min. Statistical parameters such as total movement distance, average movement speed and activity mode ratio within 10 min were counted.

### Preparation of cortico-striatal slices

After fatiguing exercise, mice were anesthetized with isoflurane (5%, 0.8 L/min) and decapitated. The brains were immediately removed and chilled in ice-cold modified artificial cerebrospinal fluid (mACSF) containing (in mM): 213 Sucrose, 2.5 KCl, 1.25 NaH_2_PO_4_, 26 NaHCO_3_, 10 D-glucose, 2 MgSO_4_ and 2 CaCl_2_ Coronal corticostriatal Slices (300 mm thick for whole-cell recording) were cut with a vibratome (VT1000S, Leica, Germany) in ice-cold mACSF. Fresh slices were then transferred to a chamber containing regular artificial cerebrospinal fluid (in mM, 126 NaCl, 2.5 KCl, 1.25 NaH_2_PO_4_, 26 NaHCO_3_, 25 D-glucose, 2 MgSO_4_ and 2 CaCl_2_) at 30°C for at least 1 h before recording. All solutions were saturated with 95% O_2_/5% CO_2_.

### Whole-cell patch clamp recording

The candidate MSNs in dorsolateral striatum were identified by using infrared-differential interference contrast video microscopy (BX50WI, Olympus, Japan). Patch pipettes (3–5 MΩ) were made from borosilicate glass capillaries pulled on a P-97 micropipette puller (Sutter Instruments, Novato, CA, USA). Whole-cell patch clamp recordings were performed in gap-free acquisition mode with a sampling rate of 10 kHz an low-pass filtered at 2 kHz, using MultiClamp 700B amplifier, Digidata 1550 and pClamp 10.5 software (Molecular Devices, Sunnyvale, CA, USA). Access resistance was continuously monitored during the experiments. Cells were excluded if the access resistance was >25 MΩ.

Action potentials were elicited by injection of a series of hyperpolarizing and depolarizing current steps (500 ms, from −100 to + 500 pA in 20 pA steps). The resting membrane potential was recorded under the “gap-free” model, and the average of at least 5-min steady membrane potential was used for statistical analysis. Input resistance was calculated as the ratio of the peak voltage to the injection current at the 40 pA (Ma et al., 2018). MSNs were identified by their medium-sized somas and characteristic electrophysiological properties, including hyperpolarized resting membrane potential, inward rectification in the hyperpolarizing direction, delayed action potential discharge with respect to onset of current injection, and low input resistance (Pawlak and Kerr, 2008). For spontaneous excitatory postsynaptic current (sEPSC) recording, MSNs were voltage-clamped at −70 mV in the presence of picrotoxin (50 μM). Internal electrode fluid for resting membrane potential, action potential and sEPSC recording contained (in mM) 140 K gluconate, 3 KCl, 2 MgCl_2_, 0.2 EGTA, 10 HEPES, 2 ATP (Na^+^ salt), with pH adjusted to 7.2 by KOH, and osmotic pressure adjusted to 280–290 mOsmol/L.

For Paired-pulse radio (PPR) recording, stimulating electrodes was placed in the white matter, and PPR was elicited by paired stimulus with various inter-stimulus intervals (ISI; 20, 50, 100, 200, and 500 ms), and calculated as the ratio of the second EPSC amplitude to the first EPSC (EPSC2/EPSC1). The internal electrode fluid contained (in mM) 120 CsMeSO3, 15 CsCl, 8 NaCl, 10 TEA, 10 HEPES, 5 QX-314, 0.2 EGTA, 2 ATP (Mg^2+^ salt), 0.3 GTP (Na^+^ salt). The PH was adjusted to 7.3 by CsOH, and osmotic pressure adjusted to 290–300 mOsmol/L.

### Statistical analysis

All numerical data were expressed as mean ± SEM. Student’s *t*-test was used to determine the statistical significance between two group means, one-way ANOVA was used to determine the statistical significance among three group means. sEPSC data were analyzed by using Mini Analysis software (Synaptosoft, Fort Lee, NJ, USA), with an amplitude threshold of 5 pA. Cumulative distributions were generated by using sEPSCs from each recording (5 min) (Peng et al., 2009; Ma et al., 2015). Kolmogorov–Smirnov test was used to compare cumulative distributions of sEPSC. The significance level was set at *p* < 0.05.

## Results

### TH-immunoreactivity and histological results

**FIGURE 3:**
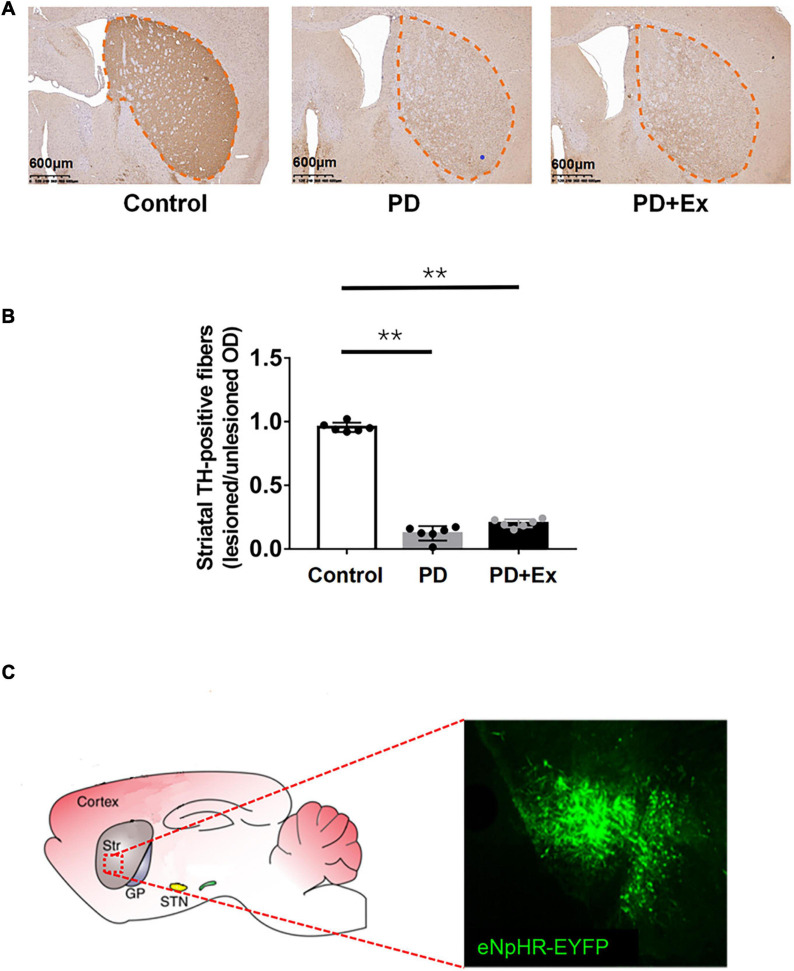
Striatal tyrosine hydroxylase (TH) positive fiber expression. **(A)** Schematic diagram of TH staining of mice in deach group (black outline, scale bar = 1.25 mm); **(B)** the ratio of TH positive fiber expression; **(C)** eNpHR-EYFP expression in Str. Micrographs of a brain slice in confocal scanning (one-way ANOVA, *p* < 0.05); all data are showed as mean ± SEM. **p < 0.01.

The results of immunohistochemistry showed that the TH-positive fibers in the bilateral striatum of the Control group were uniform, dense and symmetrical (as shown in Figure 3). The PD-positive (right) striatum TH-positive fibers were lost in the PD and PD + Ex groups, and bilateral asymmetry appeared. The ratio of TH-positive fibers on the injured and uninjured sides of the striatum in PD group was significantly lower than that in the Control group (Figure 3B, *p* < 0.05). There was no significant difference between the PD and PD + Ex groups (Figure 3B, *p* > 0.05). The results of confocal scanning showed that D2-MSNs showed obvious green fluorescence under the excitation light (Figure 3C).

### Effects of exercise on the excitability of striatum D2-medium spiny neurons

**FIGURE 4:**
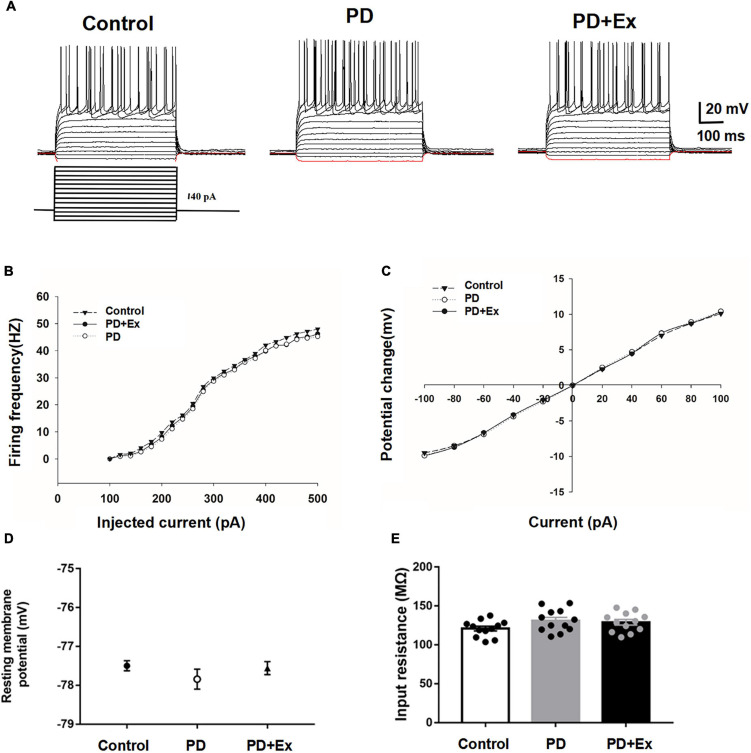
Basic electrophysiological properties of D2-MSNs in each group. **(A)** Sample traces obtained from a MSN in response to injection of hyperpolarizing and depolarizing current pulses. The firing rate vs. injected current **(B)** and I–V curves **(C)** were indistinguishable among the three groups. Resting membrane potential **(D)** and input resistance **(E)** were both comparable among the three groups (one-way ANOVA, *p* > 0.05). All data are showed as mean ± SEM.

Striatum D2-MSNs exhibited typical electrophysiological characteristics such as inward rectification and action potential release delay (Figure 4A). There was no significant difference in the frequency of action potential and I-V curve between the striatum D2-MSNs in each group (Figures 4B,C, *p* > 0.05). The resting membrane potential of D2-MSNs results showed that there was no significant difference between the groups (Figure 4D, Control: −77.50 ± 0.13 mV, *n* = 12 cells; PD −77.84 ± 0.26 mV, *n* = 12 cells; PD + Ex: −77.55 ± 0.17 mV, *n* = 12 cells, *p* > 0.05). The input impedance results showed that there was no significant difference in the input impedance between the groups (Figure 4E, Control: 120.89 ± 3.03 MΩ, *n* = 12 cells; PD: 131.05 ± 4.18 MΩ, *n* = 12 cells; PD + Ex: 128.95 ± 3.58 MΩ, *n* = 12 cells, *p* > 0.05).

**FIGURE 5:**
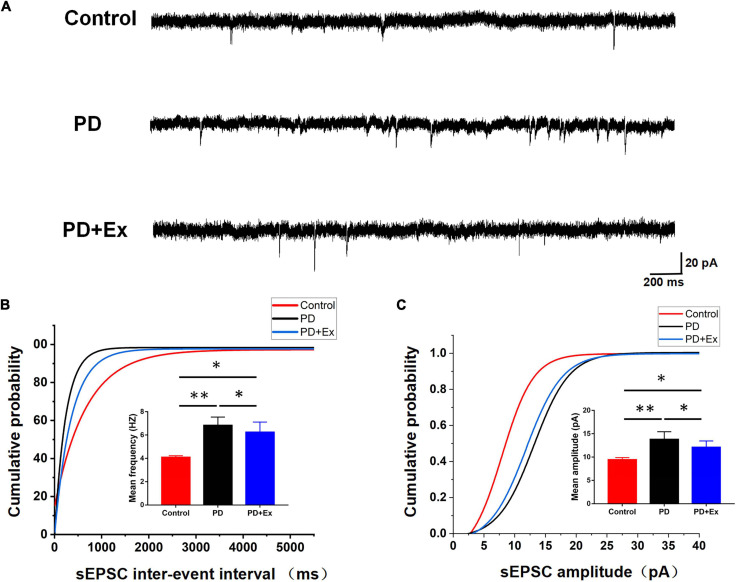
Effect of exercise on D2-MSNs sEPSC in PD mice. **(A)** Sample traces of sEPSCs recorded in D2-MSNs. **(B)** Cumulative distributions of inter-event interval and bar charts showed sEPSC frequency (Kolmogorov–Smirnov test and one-way ANOVA, respectively, *p* < 0.05). **(C)** Cumulative distributions of amplitude and bar charts showed sEPSC amplitude (Kolmogorov–Smirnov test and one-way ANOVA, respectively, *p* < 0.05. A total of 1,275 events for the Control group, 2,106 events for PD group and 1,896 events for PD + Ex group were plotted in Cumulative distributions). All data are showed as mean ± SEM. *p < 0.05, ^**^p < 0.01.

To investigate whether exercise inhibits the excitability of striatum D2-MSNs, reducing cortical-striatum synaptic transmission in PD model mice, we examined the sEPSC in each group. The sEPSC of the striatum D2-MSNs of each group was recorded by whole-cell patch clamp (as shown in Figure 5). The results showed that the average frequency of sEPSC in the PD group was significantly higher than that in the Control group, the average frequency of sEPSC in PD + Ex group was significantly lower than that in PD group but higher than Control group (Figure 5B, Control: 4.08 ± 0.04 Hz, *n* = 12 cells; PD: 6.82 ± 0.21 Hz, *n* = 12 cells; PD + Ex: 6.24 ± 0.25 Hz, *n* = 12 cells, *p* < 0.01, *p* < 0.05); PD group sEPSC average amplitude was significant higher than the Control group, the average amplitude of sEPSC in the PD + Ex group was significantly lower than that of PD group and was higher than the Control group (Figure 5C, Control: 9.40 ± 0.14 pA, *n* = 12 cells; PD: 13.78 ± 0.47 pA, *n* = 12 cells; PD + Ex: 12.09 ± 0.39 pA, *n* = 12 cells, *p* < 0.01, *p* < 0.05). In addition, the results showed that the cumulative frequency distribution curve of the sEPSC in PD group was significantly shifted to the left compared with the Control group. The cumulative frequency distribution curve of the sEPSC PD + Ex group was significantly shifted to the right compared with the PD group (Figure 5B); The cumulative amplitude distribution curve of the sEPSC in PD group was significantly shifted to the left compared with the Control group, and cumulative amplitude distribution curve of the sEPSC in PD + Ex group was significantly shifted to the right compared with the PD group (Figure 5C).

**FIGURE 6:**
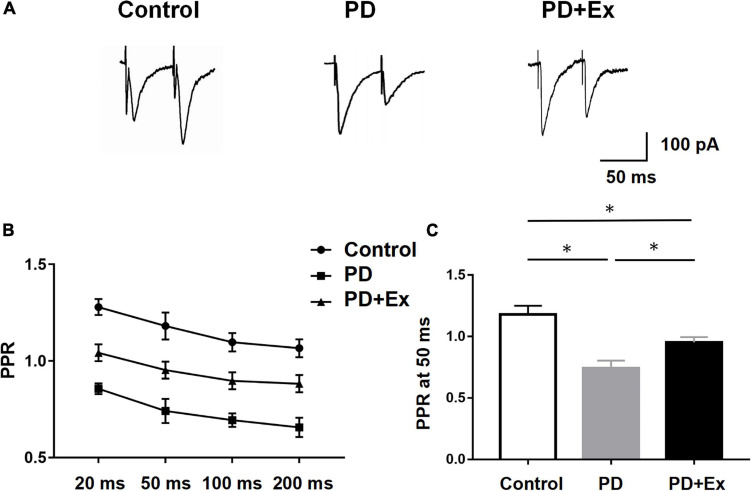
Effect of exercise on D2-MSNs PPR in PD mice. **(A)** Representative traces of EPSCs evoked by paired-pulse stimulation at 50 inter-stimulus intervals (ISI). **(B)** Summary graph of PPR from MSNs (stimulation at 20, 50, 100, and 200 ms inter-stimulus intervals, two-way ANOVA, *p* < 0.05). **(C)** PPR at 50 ms interval in each group (one-way ANOVA, *p* < 0.05). All data are showed as mean ± SEM. *p < 0.05.

To further verify that the striatal presynaptic Glu release was increased after exercise, we also examined the PPR of D2-MSNs (Figure 6A). In this experiment, the time intervals between 4 sets of double pulses were selected, which were 20, 50, 100, and 200 ms, respectively. When the time interval between double pulses was 50 ms, the PPR of striatum D2-MSNs in each group appeared significant difference. PPR of the PD group was significantly lower than that of the Control group, the PPR of the PD + Ex group was significantly higher than that of the PD group (Figure 6, Control: 1.18 ± 0.07, *n* = 10 cells; PD: 0.74 ± 0.06, *n* = 8 cells; PD + Ex: 0.95 ± 0.04, *n* = 8 cells, *p* < 0.05).

**FIGURE 7:**
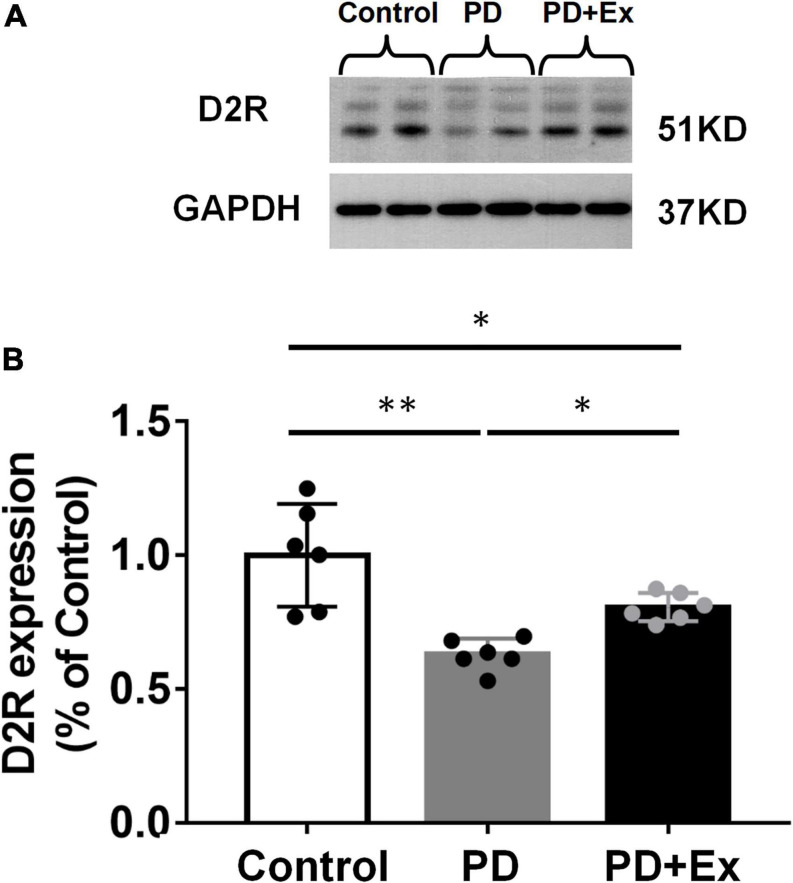
Effect of exercise on the expression of striatal D2R in PD mice. **(A)** Striatum D2R protein expression western blot. **(B)** Striatum D2R expression in each group (one-way ANOVA, *p* < 0.05). All data are showed as mean ± SEM. *p < 0.05, ^**^p < 0.01.

Western blot results showed that the D2R protein expression level in the PD group was significantly down-regulated compared with the Control group (*p* < 0.01); compared with the PD group, the expression level of D2R protein in the striatum in PD + Ex group was significantly up-regulated, but still significantly lower than that in the Control group (Figure 7, *p* < 0.05).

### Exercise and optogenetic-stimulation of D2-medium spiny neurons improved open field test

**FIGURE 8:**
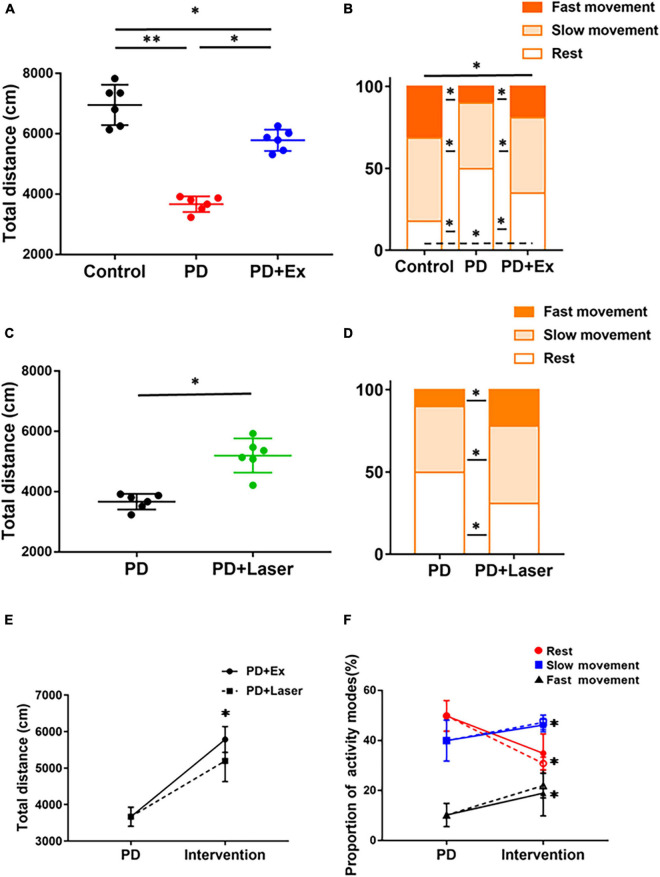
Effects of intervention on autonomic mobility in mice. Total distance **(A)** and proportion of different activity modes after exercise (one-way ANOVA, *p* < 0.05) **(B)**. Total distance **(C)** and proportion of different activity modes **(D)** after optogenetic-stimulation (Student’s *t*-test, *p* < 0.05). Comparison of autonomic activity of PD mice after exercise and optogenetic-stimulation [solid line: PD + Ex; dotted line: PD + Laser, **(E,F)**] (one-way ANOVA, *p* < 0.05). All data are showed as mean ± SEM. *p < 0.05, ^**^p < 0.01.

A comparative analysis of the effects of exercise and optogenetic-stimulation on the autonomic behavior of PD mice is shown in Figure 8. The results showed that the total movement distance in PD group was significantly reduced (*p* < 0.01, Figure 8A, compared with Control group), the percentage of resting state was significantly increased, and both in fast movement state and slow movement state were reduced significantly (*p* < 0.05, Figure 8B, compared with Control group). Compared with before intervention, the total movement distance after exercise (*p* < 0.05, Figure 8A) and optogenetic-stimulation were significantly increased (*p* < 0.05, Figure 8C); the percentage of resting state was significantly reduced, fast movement state and slow movement state increased significantly (*p* < 0.05, Figures 8E,F).

## Discussion

The goal of the present study is to examine whether exercise affects the behavioral dysfunction and excitatory synaptic transmission of D2-MSNs in PD mice. PD mice were trained to run daily for 4 weeks on an electric treadmill to establish an exercise model. Treadmill exercise can effectively decrease the amplitude and frequency of sEPSC of D2-MSNs, and increase the PPR of D2-MSNs, while leaving basic electrophysiological properties of D2-MSNs unaffected. Further behavioral results confirm that the two intervention methods of exercise and inhibiting D2-MSN by optogenetics have basically similar effects on improving the autonomic behavior of PD mice. The inhibited sEPSC of D2-MSNs may contribute to the behavioral improvement after exercise.

Medium spiny neurons act as postsynaptic neuronal cell bodies in the cortical-striatum pathway and are regulated by Glu and DA. A study has confirmed that DA loss caused a compensatory increase in the release of Glu from the cortical-striatum synaptic terminals, which was responsible for the increase in the frequency and amplitude of striatal EPSC (Lindefors and Ungerstedt, 1990). However, the changes in the basic electrophysiological properties of MSNs under pathological PD conditions remain controversial, the action potential burst in the striatal MSNs of the PD mouse model and its IV curve were not significantly different from those of normal mice (Calabresi et al., 1993). To explore whether exercise can affect the intrinsic membrane properties, we tested the action potential firing frequency, I-V relationship, resting membrane potential, and input resistance of D2-MSNs. Results shown that the basic electrophysiological properties of D2-MSNs unaffected in Control and PD group, which is consistent with the results of a study (Rylander et al., 2013). And the basic electrophysiological properties also have no significant change after exercise. Properties are dictated more by ionic conductance channels than by morphological characteristics (Zengel et al., 1985). Although several examples of activity-dependent plasticity of neural conductances have been reported for several neurone types (Krawitz et al., 2001; Baczyk et al., 2020; Ge and Dai, 2020), no such enough evidence has been presented for electrophysiological properties subjected to different levels of voluntary activity.

D2-MSN dysfunction in PD condition is mainly manifested by glutamate-mediated excitotoxicity and abnormal function of D2R (Chen et al., 2004; Garcia et al., 2010; Wei et al., 2013), leading to excessive activation of indirect pathways and dysfunction of the basal ganglia (Surmeier et al., 2007). The abnormal function of D2-MSNs may also alter the lateral inhibitory effect of the local loop of striatal MSNs, affecting the release and dispersion of neurotransmitters (Burke et al., 2017). Additionally, abnormal excitability of D2-MSNs enhances the lateral inhibitory effect on adjacent MSNs, which may inhibit basal ganglia transmission by axon projection on direct pathways and other nuclear groups in the super-direct pathway (Cazorla et al., 2014; Straub et al., 2016; Wei et al., 2017). In our study, whole-cell patch clamp recordings showed that the sEPSC frequency and amplitude was increased and the PPR was decreased in D2-MSNs of PD mice, indicating that the probability of Glu release in the striatum D2-MSNs is enhanced.

It has been shown that exercise modifies nigrostria-striatum DA and cortical-striatum Glu neurotransmission and corrects PD basal ganglia dysfunction through neuroprotection and neurorestoration (Tajiri et al., 2010; Kim et al., 2013; Toy et al., 2014). Investigating the mechanisms underlying exercise-induced improvement, we found that exercise decreased the frequency and amplitude of sEPSC and increased the PPR of D2-MSNs. This may because exercise can inhibit the overactivation of the cortical-striatal Glu pathway (Chen et al., 2017). Besides, mGluR2/3 receptors are present in high density on corticostriatal terminals, where their activation inhibits glutamate release (Conn et al., 2005). And it has been proposed that exercise significantly increased mGluR2/3 expression with 4 weeks exercise intervention, which inhibited the overactivity of the indirect pathway in PD, potentially reversing motor deficits (Shi et al., 2019). In addition to receiving Glu inputs from the cerebral cortex and thalamus, striatum also receives a massive DA inputs from substantia nigra (Bolam et al., 2000; Kreitzer and Malenka, 2008). It has been shown that abnormal DA inputs could affect corticostriatal synaptic neurotransmitter and motor behavior (Calabresi et al., 2007). In our study, the expression of D2R protein in striatum was significantly decreased under PD pathological conditions, and exercise up-regulated the expression level of D2R protein. D2R can inhibit cAMP-dependent protein kinase (PKA) through a Gi/o protein (Girault, 2012), which could inhibited the glutamate-mediated excitotoxicity in D2-MSNs. And OFT results shown that the average total movement distance of the PD and PD + Ex groups was significantly lower than that of the Control group, whereas those of the PD + Ex and PD + Laser groups were significantly higher than those of the PD group. Moreover, the total movement distance and activity mode ratio of the PD + Ex and PD + Laser groups were similar. The two intervention methods, i.e., exercise and light stimulation, had similar effects on improving the autonomic behavior of PD mice. Combined with behavioral improvement, our results indicate that the exercise-induced decrease in sEPSCs in striatal D2-MSNs is mainly related to the decrease in presynaptic Glu release in the cortical-striatum, while leaving the basic electrophysiological properties of MSNs unaffected. Decreased presynaptic Glu release and enhanced D2R function may contribute to the improvement of cortical-striatum synaptic excitotoxicity and behavioral dysfunction after exercise.

In conclusion, this study provides evidence that treadmill exercise can effectively improve motor dysfunction and inhibit the sEPSCs of D2-MSNs in PD mice. This behavioral improvement involves in inhibiting excitatory synaptic input of D2-MSNs by exercise. Besides, D2R function may hold the key to this process, and the mechanism by which D2R function changes during exercise requires further exploration.

## Data availability statement

The raw data supporting the conclusions of this article will be made available by the authors, without undue reservation.

## Ethics statement

This animal study was reviewed and approved by the Animal Ethics Committee of Soochow University and Beijing Normal University Committee for Animal Care.

## Author contributions

GZ, XL, and DQ provided the concept, designed the study, and participated in data analysis. GZ and DZ performed the experiments and drafting of the manuscript. All authors read and approved the final manuscript and agreed to be accountable for all aspects of work ensuring integrity and accuracy.
